# Trends in Human Papillomavirus–Associated Cancers — United States, 1999–2015

**DOI:** 10.15585/mmwr.mm6733a2

**Published:** 2018-08-24

**Authors:** Elizabeth A. Van Dyne, S. Jane Henley, Mona Saraiya, Cheryll C. Thomas, Lauri E. Markowitz, Vicki B. Benard

**Affiliations:** ^1^Epidemic Intelligence Service, CDC; ^2^Division of Cancer Prevention and Control, National Center for Chronic Disease Prevention and Health Promotion, CDC; ^3^Division of Viral Diseases, National Center for Immunization and Respiratory Diseases, CDC.

## Abstract

Human papillomavirus (HPV) is a known cause of cervical cancer, as well as some oropharyngeal, vulvar, vaginal, penile, and anal cancers. To assess trends, characterized by average annual percent change (AAPC), in HPV-associated cancer incidence during 1999–2015, CDC analyzed data from cancer registries covering 97.8% of the U.S. population. A total of 30,115 new cases of HPV-associated cancers were reported in 1999 and 43,371 in 2015. During 1999–2015, cervical cancer rates decreased 1.6% per year; vaginal squamous cell carcinoma (SCC) rates decreased 0.6% per year; oropharyngeal SCC rates increased among both men (2.7%) and women (0.8%); anal SCC rates also increased among both men (2.1%) and women (2.9%); vulvar SCC rates increased (1.3%); and penile SCC rates remained stable. In 2015 oropharyngeal SCC (15,479 cases among men and 3,438 among women) was the most common HPV-associated cancer. Continued surveillance through high-quality cancer registries is important to monitor cancer incidence and trends in these potentially preventable cancers.

HPV causes cervical cancer and some types of oropharyngeal, vulvar, vaginal, penile, and anal cancer; HPV DNA is found in specific tissue types that include carcinomas of the cervix and SCCs of the vulva, vagina, penis, oropharynx, and anus (*1*,*2*). The natural history from HPV infection to precancerous lesion to invasive cervical cancer is well established. HPV is the most commonly sexually transmitted infection in the United States and is often acquired soon after initiating sexual activity (*3*). Studies indicate that approximately 90% of new cervical HPV infections, including types that cause cancer, clear or become undetectable within 2 years, and those that do not clear take decades to progress to invasive cervical cancer.* Less is known about carcinogenic progression of HPV-associated infection at other anatomic sites (*2*).

CDC analyzed data from population-based cancer registries that participate in the CDC’s National Program of Cancer Registries and the National Cancer Institute’s Surveillance, Epidemiology, and End Results program that met the criteria for high data quality for all years from 1999 to 2015; these data cover approximately 97.8% of the U.S. population.^†^ Invasive cancers are not tested for HPV in most cancer registries; therefore, an HPV-associated cancer was defined as an invasive malignancy in which HPV DNA was frequently found in special studies, including carcinomas of the cervix (i.e., SCC, adenocarcinomas, and other carcinomas) and SCC of the vulva, vagina, penis, oropharynx, and anus (including rectal SCC) (*2*) and was microscopically confirmed.^§^ Cases were classified by anatomic site and cell type using the *International Classification of Diseases for Oncology, Third Edition*. Oropharyngeal SCC included squamous cell cancer types at the base of tongue, pharyngeal tonsils, anterior and posterior tonsillar pillars, glossotonsillar sulci, anterior surface of soft palate and uvula, and lateral and posterior pharyngeal walls. Anal SCC also included rectal SCCs because they are biologically similar and might be misclassified. Age-adjusted incidence rates were calculated per 100,000 persons and standardized to the 2000 U.S. standard population. Trends were measured with AAPC in rates calculated using joinpoint regression.^¶^ Rates were considered to increase if the AAPC was greater than zero (p<0.05) and to decrease if the AAPC was less than zero (p<0.05); otherwise, rates were considered stable. A maximum of two joinpoints was used. Rates and trends were estimated by sex, age group, race,** ethnicity,^††^ and region.^§§^

In the United States, a total of 30,115 new cases of HPV-associated cancer were reported in 1999 and 43,371 in 2015 (Table 1). In 1999, cervical carcinoma (13,125 cases) was the most common HPV-associated cancer: 3,750 more cases of cervical carcinoma than of oropharyngeal SCC were identified. During 1999–2015 cervical carcinoma rates decreased 1.6% per year, and oropharyngeal SCC rates increased 2.7% per year among men and 0.8% per year among women (Figure 1) (Figure 2). In 2015, there were 11,788 reported cases of cervical carcinoma and 18,917 cases of oropharyngeal SCC, including 15,479 (82%) among men and 3,438 (18%) among women.

**TABLE 1:** Annual number and annual age-adjusted rates* and trends^†^ in HPV-associated cancer,^§^ by sex, cancer type, and age group — United States,^¶^ 1999–2015

Cancer type/Age group (yrs)	Period of diagnosis		
	1999	2015	1999–2015
	No. (rate)*	No. (rate)*	AAPC (95% CI)
Total	30,115 (11.2)	43,371 (12.1)	0.5^†^ (0.2 to 0.9)
**Females**			
**All HPV-associated cancers**	**21,008 (14.6)**	**24,432 (13.6)**	**-0.4^†^ (-0.7 to 0.2)**
**Cervical carcinoma**	**13,125 (9.3)**	**11,788 (7.2)**	**-1.6^†^ (-2.2 to -1.0)**
15–19	21 (0.2)	—**	—**
20–24	161 (1.8)	74 (0.7)	-4.2^†^ (-5.3 to -3.1)
25–29	677 (7.1)	535 (5.0)	-2.6^†^ (-3.7 to -1.5)
30–34	1,252 (12.5)	1,069 (10.1)	-1.2^†^ (-2.1 to -0.4)
35–39	1,663 (14.8)	1,296 (13.0)	-0.8 (-2.1 to 0.4)
40–44	1,799 (16.4)	1,531 (15.4)	-0.8^†^ (-1.2 to -0.3)
45–49	1,571 (16.1)	1,436 (14.0)	-1.2^†^ (-2.2 to -0.2)
50–54	1,266 (15.0)	1,315 (11.8)	-1.6^†^ (-2.3 to -0.8)
55–59	1,095 (16.6)	1,292 (11.8)	-2.0^†^ (-2.9 to -1.2)
60–64	877 (16.0)	1,053 (10.8)	-2.7^†^ (-3.4 to -1.9)
65–69	772 (15.4)	808 (9.8)	-2.9^†^ (-3.4 to -2.4)
≥70	1,970 (13.2)	1,377 (7.9)	-3.2^†^ (-3.8 to -2.6)
**Vulvar SCC**	**2,615 (1.7)**	**3,890 (2.0)**	**1.3^†^ (1.1 to 1.6)**
<40	192 (0.2)	154 (0.2)	-0.5 (-1.3 to 0.3)
40–49	354 (1.7)	385 (1.9)	0.3 (-0.6 to 1.2)
50–59	361 (2.4)	778 (3.5)	2.9^†^ (2.4 to 3.4)
60–69	396 (3.8)	905 (5.0)	2.4^†^ (2.0 to 2.9)
≥70	1,312 (8.6)	1,668 (9.1)	0.5 (-0.2 to 1.2)
**Vaginal SCC**	**730 (0.5)**	**809 (0.4)**	**-0.6^†^ (-1.1 to -0.1)**
<40	28 (0)	20 (0.0)	-2.8^†^ (-4.3 to -1.2)
40–49	84 (0.4)	64 (0.3)	-0.4 (-1.5 to 0.8)
50–59	110 (0.7)	136 (0.6)	-0.2 (-1.0 to 0.6)
60–69	144 (1.4)	219 (1.2)	-0.5 (-1.6 to 0.5)
≥70	364 (2.4)	370 (2.0)	-0.6^†^ (-1.0 to -0.2)
**Anal SCC**	**2,129 (1.5)**	**4,507 (2.2)**	**2.9^†^ (2.5 to 3.3)**
<40	91 (0.1)	89 (0.1)	-1.2 (-2.5 to 0.1)
40–49	379 (1.8)	377 (1.8)	0.4 (-0.6 to 1.4)
50–59	426 (2.8)	1,347 (6.0)	4.6^†^ (3.7 to 5.6)
60–69	434 (4.1)	1,476 (8.2)	4.8^†^ (4.4 to 5.3)
≥70	799 (5.3)	1,218 (6.9)	2.1^†^ (1.7 to 2.4)
**Oropharyngeal SCC**	**2,409 (1.6)**	**3,438 (1.7)**	**0.8^†^ (0.5 to 1.1)**
<40	66 (0.1)	71 (0.1)	1 (-0.2 to 2.2)
40–49	262 (1.3)	305 (1.5)	0.4 (-0.8 to 1.6)
50–59	550 (3.6)	1,046 (4.6)	2.1^†^ (1.5 to 2.6)
60–69	653 (6.2)	1,050 (5.8)	0.4 (-0.1 to 1.0)
≥70	878 (5.9)	966 (5.6)	0.3 (-0.3 to 0.8)
**Males**			
**All HPV-associated cancers**	**9,107 (7.4)**	**18,939 (10.5)**	**2.4^†^ (2.2 to 2.6)**
**Penile SCC**	**973 (0.8)**	1,224 (0.8)	**-0.2 (-0.6 to 0.3)**
<40	40 (0.1)	34 (0.0)	-0.7 (-2.1 to 0.8)
40–49	95 (0.5)	99 (0.5)	0.6 (-0.4 to 1.6)
50–59	180 (1.3)	210 (1.0)	-1.4^†^ (-2.4 to -0.5)
60–69	242 (2.6)	287 (1.8)	-2.0^†^ (-2.6 to -1.4)
≥70	416 (4.5)	594 (4.6)	0.8^†^ (0.2 to 1.4)
**Anal SCC**	**1,168 (1.0)**	**2,236 (1.3)**	**2.1^†^ (1.4 to 2.8)**
<40	129 (0.2)	103 (0.1)	-2.9^†^ (-4.1 to -1.6)
40–49	262 (1.3)	303 (1.5)	0.8 (-0.3 to 1.9)
50–59	246 (1.7)	678 (3.2)	4.0^†^ (3.2 to 4.8)
60–69	214 (2.3)	610 (3.7)	2.7^†^ (1.9 to 3.5)
≥70	317 (3.2)	542 (4.2)	1.5 (-0.7 to 3.8)
**Oropharyngeal SCC**	**6,966 (5.6)**	**15,479 (8.5)**	**2.7^†^ (2.5 to 2.9)**
<40	147 (0.2)	133 (0.2)	-0.9 (-2.3 to 0.4)
40–49	1,217 (6.0)	1,387 (6.7)	0.8^†^ (0.2 to 1.5)
50–59	2,224 (15.6)	5,106 (23.7)	2.7^†^ (2.2 to 3.2)
60–69	1,891 (20.4)	5,745 (35.2)	4.0^†^ (3.6 to 4.3)
≥70	1,487 (14.9)	3,108 (23.1)	2.8^†^ (2.3 to 3.4)

**Sources:** CDC’s National Program of Cancer Registries and the National Cancer Institute’s Surveillance, Epidemiology, and End Results program.

**Abbreviations:** AAPC = average annual percent change; CI = confidence interval; SCC = squamous cell carcinoma.

* Per 100,000 persons, age-adjusted to the 2000 U.S. standard population.

^†^ Significant at p<0.05. Trends were measured with AAPC in rates and were considered to increase or decrease if p<0.05; otherwise rates were considered stable.

^§^ HPV-associated cancers were defined as cancers at specific anatomic sites with specific cell types in which HPV DNA frequently is found. All cancers were microscopically confirmed. Cervical cancers (*International Classification of Diseases for Oncology*, *Third Edition* [ICD-O-3] site codes C53.0–C53.9) are limited to carcinomas (ICD-O-3 histology codes 8010–8671, 8940–8941). Vaginal (ICD-O-3 site code C52.9), vulvar (ICD-O-3 site codes C51.0–C51.9), penile (ICD-O-3 site codes C60.0–60.9), anal (including rectal SCC; ICD-O-3 site code C20.9, C21.0–C21.9), and oropharyngeal (ICD-O-3 site codes C01.9, C02.4, C02.8, C05.1, C05.2, C09.0, C09.1, C09.8, C09.9, C10.0, C10.1, C10.2, C10.3, C10.4, C10.8, C10.9, C14.0, C14.2 and C14.8) cancer sites are limited to squamous cell carcinomas (ICD-O-3 histology codes 8050–8084, 8120–8131).

^¶^ Cancer incidence compiled from cancer registries that meet the data quality criteria for all invasive cancer sites combined (covering approximately 97.8% of the U.S. population).

** Data suppressed for rates when the number of cases was <6 in a year.

**FIGURE 1:**
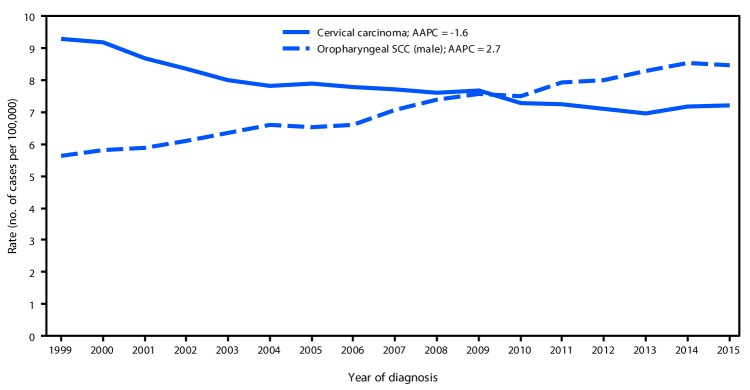
Trends* in age-adjusted incidence of cervical carcinoma among females and oropharyngeal SCC among men,^†^ — United States,^§^ 1999–2015 **Sources:** CDC's National Program of Cancer Registries; National Cancer Institute's Surveillance, Epidemiology, and End Results program. **Abbreviations**: AAPC=average annual percent change; NS=not significant; SCC = squamous cell carcinoma. * Trends were measured with AAPC in age-adjusted rates, and were considered to increase or decrease if p<0.05; otherwise trends were considered stable. ^†^ HPV-associated cancers were defined as cancers at specific anatomic sites with specific cell types in which HPV DNA frequently is found. All cancers were microscopically confirmed. Cervical cancers (*International Classification of Diseases for Oncology, Third Edition* [ICD-O-3] site codes C53.0–C53.9) are limited to carcinomas (ICD-O-3 histology codes 8010–8671, 8940–8941). Oropharyngeal (ICD-O-3 site codes C01.9, C02.4, C02.8, C05.1, C05.2, C09.0, C09.1, C09.8, C09.9, C10.0, C10.1, C10.2, C10.3, C10.4, C10.8, C10.9, C14.0, C14.2 and C14.8) cancer sites are limited to squamous cell carcinomas (ICD-O-3 histology codes 8050–8084, 8120–8131). ^§^ Cancer incidence compiled from cancer registries that meet the data quality criteria for all invasive cancer sites combined for each year during the period 1999–2015 (covering 97.8% of the U.S. population).

**FIGURE 2:**
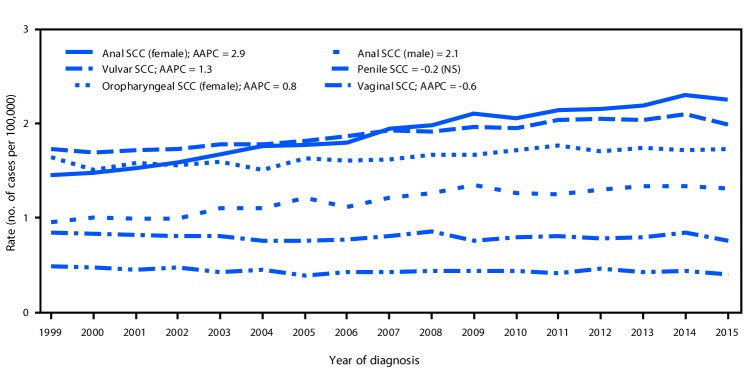
Trends* in age-adjusted HPV-associated cancer incidence,^†^ by cancer type and sex — United States,^§^ 1999–2015 **Sources:** CDC's National Program of Cancer Registries; National Cancer Institute's Surveillance, Epidemiology, and End Results program. **Abbreviations:** AAPC=average annual percent change; HPV = human papillomavirus; NS=not significant; SCC = squamous cell carcinoma. * Trends were measured with AAPC in age-adjusted rates, and were considered to increase or decrease if p<0.05; otherwise trends were considered stable. ^†^ HPV-associated cancers were defined as cancers at specific anatomic sites with specific cell types in which HPV DNA frequently is found. All cancers were microscopically confirmed. Vaginal (*International Classification of Diseases for Oncology, Third Edition* [ICD-O-3] site code C52.9), vulvar (ICD-O-3 site codes C51.0–C51.9), penile (ICD-O-3 site codes C60.0–60.9), anal (including rectal SCC; ICD-O-3 site code C20.9, C21.0–C21.9), and oropharyngeal (ICD-O-3 site codes C01.9, C02.4, C02.8, C05.1, C05.2, C09.0, C09.1, C09.8, C09.9, C10.0, C10.1, C10.2, C10.3, C10.4, C10.8, C10.9, C14.0, C14.2 and C14.8) cancer sites are limited to squamous cell carcinomas (ICD-O-3 histology codes 8050–8084, 8120–8131). ^§^ Cancer incidence compiled from cancer registries that meet the data quality criteria for all invasive cancer sites combined for each year during the period 1999–2015 (covering 97.8% of the U.S. population).

Rates of oropharyngeal SCC increased among men in all age groups ≥40 years, ranging from 0.8% among men aged 40–49 years to 4.0% among those aged 60–69 years (Table 1). Rates varied by race, with the largest increase occurring among white men (3.3%), and by region, with rates increasing more in the Midwest (3.2%) than in other regions (Table 2).

**TABLE 2:** Annual number, annual age-adjusted rate,* and trends^†^ of HPV-associated cancer cases,^§^ by sex, race, ethnicity,^¶^ and U.S. census region** — United States,^††^ 1999–2015

Cancer type/Characteristic	1999	2015	1999–2015
	No. (rate)	No. (rate)	AAPC (95% CI)
**Females**			
**Cervical carcinoma**			
**Race**			
White	10,316 (8.8)	9,079 (7.1)	-1.5^†^ (-2.0 to -1.0)
Black	2,114 (13.4)	1,731 (7.9)	-3.2^†^ (-3.8 to -2.5)
AI/AN	96 (8.8)	117 (5.9)	-1.7^†^ (-2.7 to -0.6)
API	442 (8.7)	616 (5.8)	-2.9^†^ (-3.4 to -2.4)
**Ethnicity**			
Non-Hispanic	11,293 (8.8)	9,706 (7.0)	-1.5^†^ (-1.9 to -1.0)
Hispanic	1,831 (15.2)	2,082 (8.9)	-3.4^†^ (-3.9 to -2.9)
**Region**			
Northeast	2,571 (8.8)	2,140 (6.9)	-1.7^†^ (-2.0 to -1.4)
Midwest	2,901 (8.9)	2,498 (7.3)	-1.7^†^ (-2.3 to -1.0)
South	4,935 (10.1)	4,601 (7.7)	-1.7^†^ (-2.1 to -1.3)
West	2,718 (8.8)	2,549 (6.6)	-2.0^†^ (-2.6 to -1.4)
**Vulvar SCC**			
**Race**			
White	2,394 (1.8)	3,485 (2.2)	1.5^†^ (1.3 to 1.7)
Black	186 (1.2)	289 (1.3)	1.0^†^ (0.2 to 1.8)
AI/AN	7 (0.7)	20 (1.1)	—^§§^
API	20 (0.5)	46 (0.5)	0.5 (-1.3 to 2.3)
**Ethnicity**			
Non-Hispanic	2,510 (1.8)	3,679 (2.1)	1.6^†^ (1.3 to 1.8)
Hispanic	105 (1.2)	211 (1.1)	-0.1 (-0.8 to 0.6)
**Region**			
Northeast	618 (1.9)	847 (2.2)	1.5^†^ (1.1 to 2.0)
Midwest	687 (1.9)	1,024 (2.4)	1.5^†^ (0.5 to 2.5)
South	855 (1.7)	1,366 (2.0)	1.3^†^ (1.0 to 1.7)
West	455 (1.5)	653 (1.5)	0.3 (-0.2 to 0.9)
**Vaginal SCC**			
**Race**			
White	583 (0.4)	638 (0.4)	-0.3 (-0.9 to 0.3)
Black	125 (0.9)	124 (0.6)	-2.7^†^ (-4.0 to -1.4)
AI/AN	—^§§^	—^§§^	—^§§^
API	14 (0.4)	29 (0.3)	-2.1 (-4.6 to 0.4)
**Ethnicity**			
Non-Hispanic	682 (0.5)	724 (0.4)	-0.5 (-1.0 to 0.1)
Hispanic	48 (0.5)	85 (0.4)	-1.7^†^ (-2.8 to -0.6)
**Region**			
Northeast	148 (0.5)	154 (0.4)	-0.7 (-1.6 to 0.3)
Midwest	165 (0.5)	161 (0.4)	-0.1 (-1.1 to 1.0)
South	297 (0.6)	327 (0.5)	-0.9^†^ (-1.7 to -0.1)
West	120 (0.4)	167 (0.4)	-0.5 (-1.4 to 0.3)
**Anal SCC**			
**Race**			
White	1,904 (1.5)	4,010 (2.4)	3.2^†^ (2.8 to 3.6)
Black	175 (1.2)	378 (1.6)	2.2^†^ (1.4 to 2.9)
AI/AN	8 (1.0)	22 (1.1)	—^§§^
API	21 (0.5)	47 (0.4)	-0.9 (-3.5 to 1.8)
**Ethnicity**			
Non-Hispanic	2,001 (1.5)	4,192 (2.4)	3.2^†^ (2.8 to 3.6)
Hispanic	128 (1.4)	315 (1.5)	0.5 (-0.1 to 1.2)
**Region**			
Northeast	374 (1.2)	855 (2.2)	4.3^†^ (2.7 to 6.0)
Midwest	465 (1.3)	979 (2.3)	3.6^†^ (3.0 to 4.1)
South	815 (1.6)	1,689 (2.3)	2.6^†^ (2.1 to 3.0)
West	475 (1.6)	984 (2.1)	2.2^†^ (1.6 to 2.8)
**Oropharyngeal SCC**			
**Race**			
White	2,090 (1.7)	3,022 (1.9)	1.2^†^ (0.9 to 1.5)
Black	282 (1.9)	303 (1.3)	-1.7^†^ (-2.3 to -1.2)
AI/AN	7 (0.8)	15 (0.9)	—^§§^
API	22 (0.5)	56 (0.5)	0.9 (-0.8 to 2.6)
**Ethnicity**			
Non-Hispanic	2,306 (1.7)	3,251 (1.8)	1.0^†^ (0.7 to 1.2)
Hispanic	103 (1.1)	187 (0.9)	0.1 (-1.1 to 1.3)
**Region**			
Northeast	524 (1.7)	659 (1.8)	1.1^†^ (0.6 to 1.7)
Midwest	536 (1.6)	813 (1.9)	1.5^†^ (1.0 to 2.0)
South	887 (1.8)	1,348 (1.9)	0.7^†^ (0.4 to 1.0)
West	462 (1.5)	618 (1.4)	-0.2 (-0.5 to 0.1)
**Males**			
**Penile SCC**			
**Race**			
White	864 (0.9)	1,045 (0.8)	-0.1 (-0.7 to 0.4)
Black	71 (0.7)	124 (0.8)	-0.2 (-1.3 to 0.9)
AI/AN	10 (2.1)	8 (0.5)	—^§§^
API	15 (0.5)	27 (0.3)	-0.9 (-3.3 to 1.5)
**Ethnicity**			
Non-Hispanic	865 (0.8)	1,029 (0.7)	-0.3 (-0.8 to 0.2)
Hispanic	108 (1.4)	195 (1.1)	-0.9 (-2.0 to 0.2)
**Region**			
Northeast	180 (0.8)	222 (0.8)	0 (-1.0 to 0.9)
Midwest	240 (0.9)	267 (0.8)	-0.5 (-2.0 to 0.9)
South	387 (1.0)	486 (0.8)	-0.8 (-1.9 to 0.3)
West	166 (0.7)	249 (0.7)	0.2 (-0.6 to 0.9)
**Anal SCC**			
**Race**			
White	1,008 (0.9)	1,870 (1.3)	2.1^†^ (1.6 to 2.5)
Black	136 (1.1)	315 (1.7)	3.0^†^ (1.2 to 4.7)
AI/AN	—^§§^	12 (0.6)	—^§§^
API	7 (0.2)	16 (0.2)	1.0 (-1.5 to 3.5)
**Ethnicity**			
Non-Hispanic	1,093 (1.0)	2,072 (1.4)	2.3^†^ (1.6 to 3.1)
Hispanic	75 (0.8)	164 (0.8)	-0.1 (-1.1 to 1.0)
**Region**			
Northeast	246 (1.0)	485 (1.6)	2.7^†^ (2.0 to 3.4)
Midwest	230 (0.8)	400 (1.1)	2.7^†^ (1.9 to 3.4)
South	431 (1.0)	884 (1.4)	2.3^†^ (1.6 to 3.0)
West	261 (1.0)	467 (1.2)	0.8 (-0.1 to 1.6)
**Oropharyngeal SCC**			
**Race**			
White	5,871 (5.5)	13,979 (9.2)	3.3^†^ (3.1 to 3.6)
Black	944 (8.3)	1,135 (6.1)	-1.6^†^ (-2.0 to -1.2)
AI/AN	36 (4.7)	82 (4.4)	2.5^†^ (0.9 to 4.1)
API	78 (2.0)	166 (1.9)	1.1^†^ (0.1 to 2.1)
**Ethnicity**			
Non-Hispanic	6,635 (5.8)	14,728 (9.1)	3.0^†^ (2.8 to 3.2)
Hispanic	330 (4.2)	751 (4.1)	0.1 (-0.5 to 0.7)
**Region**			
Northeast	1,367 (5.5)	2,710 (8.0)	2.6^†^ (2.4 to 2.9)
Midwest	1,569 (5.4)	3,423 (8.5)	3.2^†^ (2.9 to 3.6)
South	2,677 (6.3)	6,183 (9.4)	2.5^†^ (2.3 to 2.8)
West	1,353 (5.1)	3,163 (7.4)	2.6^†^ (2.3 to 2.9)

**Sources:** CDC’s National Program of Cancer Registries and the National Cancer Institute’s Surveillance, Epidemiology, and End Results program.

**Abbreviations:** AAPC = average annual percent change; AI/AN = American Indian/Alaska Native; API = Asian or Pacific Islander; CI = confidence interval; SCC = squamous cell carcinoma.

* Per 100,000 persons, age-adjusted to the 2000 U.S. standard population.

^†^ Significant at p<0.05. Trends were measured with AAPC in rates and were considered to increase or decrease if p<0.05; otherwise rates were considered stable.

^§^ HPV-associated cancers were defined as cancers at specific anatomic sites with specific cell types in which HPV DNA frequently is found. All cancers were microscopically confirmed. Cervical cancers (*International Classification of Diseases for Oncology, Third Edition* [ICD-O-3] site codes C53.0–C53.9) are limited to carcinomas (ICD-O-3 histology codes 8010–8671, 8940–8941). Vaginal (ICD-O-3 site code C52.9), vulvar (ICD-O-3 site codes C51.0–C51.9), penile (ICD-O-3 site codes C60.0–60.9), anal (including rectal SCC; ICD-O-3 site code C20.9, C21.0–C21.9), and oropharyngeal (ICD-O-3 site codes C01.9, C02.4, C02.8, C05.1, C05.2, C09.0, C09.1, C09.8, C09.9, C10.0, C10.1, C10.2, C10.3, C10.4, C10.8, C10.9, C14.0, C14.2 and C14.8) cancer sites are limited to squamous cell carcinomas (ICD-O-3 histology codes 8050–8084, 8120–8131).

^¶^ Racial categories are not mutually exclusive from Hispanic ethnicity. Rates are not presented for cases with unknown or other race or unknown ethnicity.

** *Midwest:* Illinois, Indiana, Iowa, Kansas, Michigan, Minnesota, Missouri, Nebraska, North Dakota, Ohio, and Wisconsin. *Northeast:* Connecticut, Maine, Massachusetts, New Hampshire, New Jersey, New York, Pennsylvania, Rhode Island, and Vermont. *South:* Alabama, Delaware, District of Columbia, Florida, Georgia, Kentucky, Louisiana, Maryland, North Carolina, Oklahoma, South Carolina, Tennessee, Texas, Virginia, and West Virginia. *West:* Alaska, Arizona, California, Colorado, Hawaii, Idaho, Montana, Nevada, New Mexico, Oregon, Utah, Washington, and Wyoming.

^††^ Cancer incidence compiled from cancer registries that meet the data quality criteria for all invasive cancer sites combined (covering approximately 97.8% of the U.S. population).

^§§^ Data suppressed for rates when the number of cases was <6 in a year.

During 1999–2015 cervical carcinoma rates were stable among women aged 35–39 years and decreased among women aged 20–34 years and aged ≥40 years, decreasing >3% per year among women aged 20–24 years and ≥70 years (Table 1). Cervical carcinoma rates decreased among all racial/ethnic groups, more among Hispanics than among non-Hispanics, and more in the West than in all other regions (Table 2). During 1999–2015 vaginal SCC decreased 0.6% per year.

In contrast, penile SCC rates were stable, and vulvar SCC rates increased 1.3% per year (Table 1) (Figure 2). Specifically, vulvar SCC rates increased during 1999–2015 among women aged 50–69 years, among whites (1.5%), and blacks (1.0%), and in the Northeast (1.5%), Midwestern (1.5%), and Southern (1.3%) regions of the United States.

Anal SCC rates increased among women (2.9% per year) and men (2.1%) during this period. The largest increases in anal SCC rates were among women aged 50–69 years (4.6%–4.8% per year) and men aged 50–59 years (4.0%). Anal SCC rates increased among white women (3.2% per year), black women (2.2%), white men (2.1%), and black men (3.0%). Anal SCC rates increased among both men and women in all regions except among men in the West region; the largest rate increases were among women in the Northeast (4.3% per year) and Midwest (3.6%).

## Discussion

HPV-associated cancer rates changed from 1999 to 2015. Rates increased for oropharyngeal SCC, anal SCC and vulvar SCC, decreased for cervical carcinoma and vaginal SCC, and remained stable for penile SCC.

The decline in cervical cancer from 1999 to 2015 represents a continued trend since the 1950s as a result of cancer screening (*4*). Rates of cervical carcinoma in this report decreased more among Hispanics, American Indian/Alaska Natives, and blacks than other groups; however, incidence rates were still higher among Hispanics and blacks than among whites in 2015. These persistent disparities in incidence suggest that health care delivery needs of some groups are not fully met.

Several factors could contribute to the increase in oropharyngeal and anal cancers including changing sexual behaviors. Unprotected oral sex and receptive anal sex are risk factors for HPV infection (*2*,*5*). White men have the highest number of lifetime oral sex partners and report first performing oral sex at a younger age compared with other racial/ethnic groups; these risk factors could be contributing to a higher rate of oropharyngeal SCC among white men than other racial/ethnic groups (*6*). Although smoking is a risk factor for oropharyngeal cancers, smoking rates have been declining in the United States, and studies have indicated that the increase in oropharyngeal cancer is attributable to HPV (*5*). In contrast to cervical cancer, there currently is no U.S. Preventive Services Task Force recommended screening for other HPV-associated cancers (*7*).

The findings in this report are subject to at least two limitations. First, although population-based cancer registries provide a reliable system for counting invasive cancers, registries do not routinely determine the HPV status of cancers. In the United States, HPV DNA has been determined through special studies and found in 91% of cervical, 91% of anal, 75% of vaginal, 70% of oropharyngeal, 69% of vulvar, and 63% of penile cancers (*1*). Second, reporting of race and ethnicity uses data from medical records, which might be inaccurate in a small proportion of cases. An important strength of this study is the use of high quality population-based surveillance data with 97.8% coverage of the U.S. population, allowing for specific histologic definitions to monitor HPV-associated cancer trends.

Measures to prevent HPV-associated diseases in the United States include both females and males; HPV vaccination was included in the routine immunization program for females in 2006 and for males in 2011. Although it might be too soon for effects on invasive cancers from HPV vaccination in the United States, studies have reported reductions in cervical HPV infection, genital warts, and cervical precancers (*8*). Most cervical cancers are preventable with both HPV vaccination and regular and timely screening among women aged 21–65 years with follow-up for abnormal test results. Routine HPV vaccination is recommended at age 11 or 12 years; currently, the 9-valent HPV vaccine, which targets oncogenic types attributed to 73% of HPV-associated cancers, is being used in the United States (*1*,*9*). Further research to understand the progression from HPV infection to oropharyngeal cancer would be beneficial. Continued surveillance through high-quality registries is important to monitor changes in HPV-associated cancer incidence.

**Summary**

**What is already known about this topic?**

Human papillomavirus (HPV) can cause some types of cervical, vulvar, vaginal, penile, anal, and oropharyngeal cancers.

**What is added by this report?**

Oropharyngeal squamous cell carcinoma is now the most common HPV-associated cancer. During 1999–2015 cervical carcinoma incidence rates decreased 1.6% per year, and oropharyngeal SCC incidence rates increased 2.7% per year among men and 0.8% per year among women.

**What are the implications for public health practice?**

Population-based screening is recommended for only one HPV-associated cancer (cervical) at this time; however, HPV vaccination can prevent infection with the HPV types most strongly associated with cancer. Ongoing surveillance for HPV-associated cancers using high-quality population-based registries is critical to monitor cancer rates and trends.

## Footnotes

* Manual for the Surveillance of Vaccine-Preventable Diseases, Chapter 5: Human Papillomavirus (HPV). https://www.cdc.gov/vaccines/pubs/surv-manual/chpt05-hpv.html.

† Cancer registries’ incidence data met the following five United States Cancer Statistics criteria: 1) ≤5% of cases ascertained solely on the basis of death certificate; 2) ≤3% of cases missing information on sex; 3) ≤3% of cases missing information on age; 4) ≤5% of cases missing information on race; and 5) ≥97% of registry’s records passed a set of single-field and interfield computerized edits that test the validity and logic of data components. https://gis.cdc.gov/Cancer/USCS/DataViz.html.

§ HPV-associated cancers were defined as cancers at specific anatomic sites with specific cell types in which HPV DNA frequently is found. All cancers were microscopically confirmed. Cervical cancers (ICD-O-3 http://codes.iarc.fr/ [ICD-O-3] site codes C53.0–C53.9) are limited to carcinomas (ICD-O-3 histology codes 8010–8671, 8940–8941). Vaginal (ICD-O-3 site code C52.9), vulvar (ICD-O-3 site codes C51.0–C51.9), penile (ICD-O-3 site codes C60.0–60.9), anal (including rectal SCC; ICD-O-3 site code C20.9, C21.0–C21.9), and oropharyngeal (ICD-O-3 site codes C01.9, C02.4, C02.8, C05.1, C05.2, C09.0, C09.1, C09.8, C09.9, C10.0, C10.1, C10.2, C10.3, C10.4, C10.8, C10.9, C14.0, C14.2 and C14.8) cancer sites are limited to squamous cell carcinomas (ICD-O-3 histology codes 8050–8084, 8120–8131). Anal and rectal SCC were combined into a single category “anal SCC” because a very small subset of rectal cancers (i.e. the SCCs, around 700 per year) are similar to anal SCC.

¶ https://surveillance.cancer.gov/joinpoint/.

** Population estimates incorporate bridged single-race estimates derived from the original multiple-race categories in the 2010 U.S. Census. https://seer.cancer.gov/popdata.

†† https://www.census.gov/prod/cen2010/briefs/c2010br-04.pdf.

§§ *Midwes*t: Illinois, Indiana, Iowa, Kansas, Michigan, Minnesota, Missouri, Nebraska, North Dakota, Ohio, and Wisconsin. *Northeast*: Connecticut, Maine, Massachusetts, New Hampshire, New Jersey, New York, Pennsylvania, Rhode Island, and Vermont. *South*: Alabama, Delaware, District of Columbia, Florida, Georgia, Kentucky, Louisiana, Maryland, North Carolina, Oklahoma, South Carolina, Tennessee, Texas, Virginia, and West Virginia. *West*: Alaska, Arizona, California, Colorado, Hawaii, Idaho, Montana, Nevada, New Mexico, Oregon, Utah, Washington, and Wyoming.
